# *Anopheles sundaicus* Mosquitoes as Vector for *Plasmodium knowlesi*, Andaman and Nicobar Islands, India

**DOI:** 10.3201/eid2504.181668

**Published:** 2019-04

**Authors:** Pachalil Thiruvoth Vidhya, Ittoop Pulikkottil Sunish, Anwesh Maile, Ali Khan Zahid

**Affiliations:** ICMR Regional Medical Research Centre, Port Blair, Andaman and Nicobar Islands, India

**Keywords:** *Plasmodium knowlesi*, *Anopheles sundaicus*, mosquitoes, macaques, malaria, PCR, DNA, parasites, Andaman and Nicobar Islands, vector-borne infections, India

## Abstract

Using PCR and sequencing, we found *Plasmodium knowlesi* in the malaria vector *Anopheles sundaicus* mosquito collected from Katchal Island in the Andaman and Nicobar Islands, India. We cannot rule out natural transmission of this parasite to humans through this mosquito species. An in-depth investigation is needed to prevent disease outbreaks.

*Plasmodium knowlesi* is a simian malaria parasite and is recognized as the fifth human malaria parasite (*1*). Natural infection in humans by *P. knowlesi* was first reported in Malaysian Borneo (*2*). Mosquitoes belonging to Leucosphyrus group were reported as vectors of *P. knowlesi* in Southeast Asia countries. Among this group, *Anopheles latens* mosquitoes were identified as a vector of *P. knowlesi* in Malaysian Borneo (*3*). Similar vectors have been reported elsewhere, including *An. dirus* mosquitoes in Vietnam (*4*); *An. balabacensis* mosquitoes in Sabah, Malaysia (*5*); *An. hackeri* mosquitoes in Malaya (now Peninsular Malaysia) (*6*); and *An. cracens* mosquitoes in Kuala Lipis and *An. introlatus* mosquitoes in Selangor, Malaysia (*7*). Experimental studies on the H strain of *P. knowlesi* showed that *An. balabacensis* mosquitoes were the most competent vector, followed by *An. stephensi*, *An. maculatus*, and *An. freeborni* mosquitoes (*8*). However, no studies record isolation of *P. knowlesi* from *An. sundaicus* mosquitoes of the Pyretophorus series. This anopheline species is a known vector of malaria parasites in the Andaman and Nicobar Islands, India.

In 1980, a simian malaria and *P. vivax*–like parasite, *P. cynomolgi,* was isolated from *An. sundaicus* mosquitoes and from a subspecies of macaque, *Macaca umbrosus*, at Great Nicobar (*9*). In a later study, researchers sequenced 445 archival blood samples from human malaria cases from the Andaman and Nicobar Islands and found 11.9% (53) were positive for *P. knowlesi*, both mono- and co-infections. (*10*). Investigators also suspected earlier research might have mistakenly identified *P. knowlesi* as *P. cynomolgi* because the species are difficult to distinguish by light microscopy. In view of these findings, we conducted an entomologic and parasitologic survey in selected islands of Nicobar district to identify the role of anophelines in the transmission of *P. knowlesi*. 

## The Study

The Nicobar district is an endemic area for malaria; annual parasitic incidence was 7.04–16.07/1,000 population/year during 2013–2017. In the Nicobar district, Katchal and Great Nicobar Islands are known for high malaria case counts and for macaques that frequently come close to human habitations. Long-tailed macaques, *Macaca fascicularis umbrosa*, are the only nonhuman primates found on Nicobar Islands (*11*). As yet, the malaria surveillance program has not reported human infection with *P. knowlesi* in the district.

During 2016–2018, we selected 3 villages on Katchal Island (Mildera, Japan Tickrey, and Meenakshi Ram Nagar) and 3 villages on Great Nicobar Island (Rajiv Nagar, Govindnagar, and Sastrinagar). Our selection was based on high numbers of malaria cases and prevalence of macaques. Katchal Island, population 2,658, has a hilly terrain with an area of 146.5 km^2^ and has dense tropical rain forest (Indian Census data, http://www.censusindia.gov.in/2011). Great Nicobar, population 3,500, has both plains and hilly terrain and is the largest island of Nicobar district at 921 km^2^.

We collected adult mosquitoes by night landing and light trap collections for 8 nights in each village from 5:30 pm through 5:30 am. We also collected adult mosquitoes by indoor resting collection from 5:00 am to 8:00 am. We conducted night landing collections using human, bovine, and caprine baits. We conducted resting collections with oral aspirators in and around 16 sites in each village, including human shelters and animal sheds. We installed 8 light traps in each village. We used standard taxonomic keys for morphological identification (*12*). We collected blood slides of microscopically confirmed *P. falciparum* and *P. vivax* (n = 55) from the local primary health centers and conducted assays for molecular confirmation, including for *P. knowlesi.* In addition, we tested filter paper blood spots (n = 106) that were collected through active fever surveillance and were positive by bivalent rapid diagnostic tests.

We tested 54 pools, 10 mosquitoes per pool, including 35 pools of *An. sundaicus* mosquitoes, 18 pools of *An. maculatus* mosquitoes, and 1 pool with 2 specimens of *An. barbirostris* mosquitoes*.* We extracted DNA from each pool of anophelines, and from slides and filter paper blood spots, using GeNei Whole Blood DNA Extraction Kit solution (Genie, http://geneilabs.com), according to the manufacturer’s instructions. We screened the DNA extracts for all 5 malaria parasites by nested PCR, as suggested by Singh et al. (*13*), amplifying the 18S small subunit ribosomal RNA of *P. knowlesi.* We used Pmk8 and Pmk9 primers to amplify *P. knowlesi*. We used rPLU1 and rPLU5 primers for the first step and other primers, including rFAL1and rFAL2, rVIV1 and rVIV2, rOVA1 and rOVA2, rMAL1 and rMAL2, for the second step of nested PCR. We obtained positive control *P. knowlesi* blood spots from the National Institute of Malaria Research (New Delhi, India).

**Figure 1:**
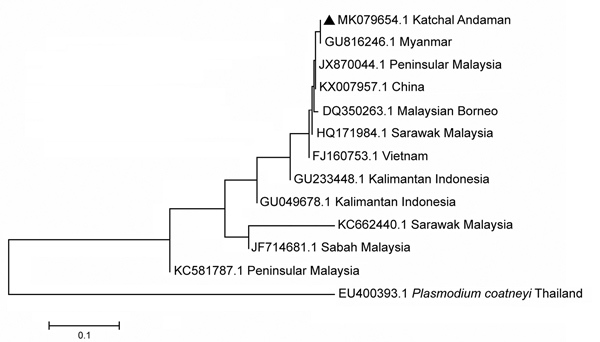
Phylogenetic relationship of *Plasmodium knowlesi* from the Andaman and Nicobar Islands, India, to other strains from Southeast Asia countries, inferred by using the neighbor-joining method. Isolates are identified by GenBank accession number and location; *Plasmodium coatneyi* is used as outgroup. Triangle indicates sequence of *P. knowlesi* isolated in this study (accession no. MK079654) that shares a similar clade with a Myanmar sequence (accession no. GU816246). Isolates from Peninsular Malaysia (accession no. JX870044) and China (accession no. KX007957) are also closer to the Andaman isolate. Scale bar indicates nucleotide substitutions per site.

Samples were visualized by agarose gel electrophoresis in 2% agarose and we excised bands of 153 bp from the positive samples, then purified these using QIAquick Gel Extraction Kit (QIAGEN, http://www.qiagen.com), according to the manufacturer’s protocol. We performed sequencing on an ABI 3730 DNA Analyzer (Applied Biosystems, https://www.thermofisher.com). We checked sequence identity with reference sequences obtained from the NCBI database and submitted processed sequences to GenBank (accession no. MK079654). We constructed the phylogenetic tree with closely similar sequences using the neighbor-joining method in MEGA7 (https://www.megasoftware.net) (Figure 1).

**Figure 2:**
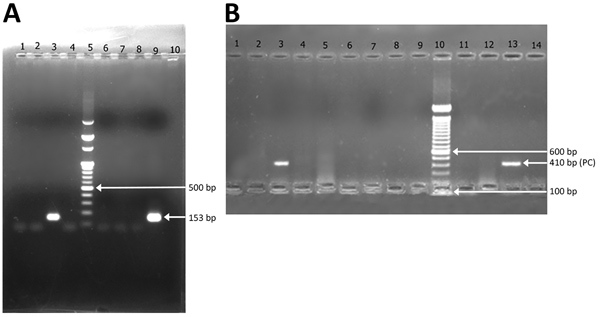
Agarose gel (2%) electrophoresis of nested PCR products of amplified *Plasmodium knowlesi* DNA sequences taken from mosquito samples (10 mosquitoes per pool) from Andaman and Nicobar Islands. A) Analysis of PCR products amplified with Pmk8 and Pmk9 primers. Lanes 1–4, pools of *Anopheles sundaicus* mosquitoes; lane 5, size marker DNA (100-bp DNA ladder); lanes 6 and 7, *An. maculatus* mosquito pools; lane 8, *An. barbirostris* mosquito pool (2 mosquitoes); lane 9, positive control; lane 10, negative control. Arrows indicate positive sample from the *An. sundaicus* mosquito pool in lane 3, 500 bp marker in DNA ladder of lane 5, and positive control in lane 9. B) Analysis of nested PCR products amplified with PKF1140 and PKR1550 primers. Lanes 1–5, *An. sundaicus* mosquito pools; lanes 6–9, *An. maculatus* mosquito pools; lane 10, size marker DNA (100-bp DNA ladder); lane 11, *An. maculatus* mosquito pool; lane 12, *An. barbirostris* mosquito pool; lane 13, positive control; lane 14, negative control. Arrows indicate *An. sundaicus* mosquito pool with positive band at 410 bp in lane 3, 600-bp marker in DNA ladder in lane 10, and positive control of 410 bp in lane 13. PC, positive control.

Of 855 mosquitoes captured, 532 were anophelines and 323 were culicines. Among anophelines, the *An.* *sundaicus* mosquito was the predominant species collected, followed by *An. maculatus* and *An. barbirostris* mosquitoes (Table). One pool of 10 of *An. sundaicus* mosquitoes collected from Mildera village on Katchal Island was found positive for *P. knowlesi* by PCR and sequencing. Five blood slide samples, 1 identified as *P. vivax* and 4 identified as co-infection with *P. falciparum* and *P. vivax* by microscopy, were found positive for *P. knowlesi* by PCR with primers Pmk8 and Pmk9 (Figure 2, panel A), but no sequences were obtained. Subsequently, these samples showed no amplification with a different set of primers, PKF1140 and PKR1550 (*14*) (Figure 2, panel B). The false amplification may be due to cross-hybridization of primers Pmk8 and Pmk9 with *P. vivax* DNA. Therefore, we could not conclude that those samples were positive for *P. knowlesi*. We found *P. knowlesi* DNA in *An. sundaicus* mosquitoes and confirmed our findings by nucleotide sequence analysis. The nucleotide sequences obtained from our study resembled *P. knowlesi* reference sequences retrieved from GenBank (accession nos. GU816246, JX870044, and KX007957). We cannot conclude that *An. sundaicus* mosquitoes are the vector of *P. knowlesi*, but our results suggest the possibility of *An. sundaicus* mosquitoes for transmission of this parasite and indicated that further in-depth investigation is warranted.

**Table:** Adult mosquitoes collected using various methods, Andaman and Nicobar Islands, India

Serial no.	Mosquito species	Light trap			Night landing, human bait			Night landing, animal bait		Total
Indoor	Outdoor	Indoor	Outdoor	Caprine	Bovine
1	*Anopheles sundaicus*	10	52		0	15		150	123	350
2	*An. maculatus*	0	0		0	10		70	100	180
3	*An. barbirostris*	2	0		0	0		0	0	2
4	*Armigeres *sp.	0	2		10	25		15	7	59
5	*Culex* sp.	2	264		0	0		0	0	264

## Conclusions

Because *P. knowlesi* is an emerging and potentially lethal malarial parasite, care and safety need to be taken to prevent the spread of infection. As van Hellemond et al. (*15*) suggest, synergistic use of *P. knowlesi*–specific rapid diagnostic tests and microscopy could identify *P. knowlesi* in health centers in inaccessible areas. An extensive parasitologic survey in humans and macaques could provide a more precise picture on the prevalence of *P. knowlesi* in the Andaman and Nicobar Islands.
